# Stapler-Less Uniportal Video-Assisted Thoracoscopic Surgery: A Case Series and Review of the Literature

**DOI:** 10.5761/atcs.oa.25-00009

**Published:** 2025-06-17

**Authors:** Rawan F. Ayyad, Alaa Ayyad, Raghad Sweity, Mayar Idkedek, Firas Abu Akar

**Affiliations:** 1 Medical Research Club, Faculty of Medicine, Al-Quds University, East Jerusalem, Palestine; 2 Department of Cardiothoracic Surgery, Makassed Charitable Society Hospital, East Jerusalem, Palestine; 3 Department of General Surgery, Faculty of Medicine, Al-Quds University, East Jerusalem, Palestine

**Keywords:** video-assisted thoracic surgery (VATS), uniportal VATS, stapler-less VATS, minimally invasive thoracic surgery

## Abstract

**Purpose:** Video-assisted thoracoscopic surgery (VATS) is a minimally invasive approach widely used for lung resections. However, reliance on staplers increases costs, limiting its adoption in resource-constrained settings. This study evaluates the feasibility, safety, and cost-effectiveness of uniportal stapler-less VATS lobectomies and segmentectomies.

**Methods:** A retrospective analysis of 7 stapler-less uniportal VATS surgeries performed between March 2021 and February 2022 was conducted. Data on operative time, blood loss, postoperative outcomes, and complications were collected from patient records.

**Results:** Seven procedures were completed with an average operative time of 80 min (range: 48–118 min). Estimated blood loss was minimal (10–100 mL) in 6 cases. One patient required conversion to open thoracotomy due to vessel injury. Postoperatively, all patients were stable with no major complications.

**Conclusion:** Stapler-less VATS is a viable, cost-effective alternative to conventional techniques, offering comparable safety and outcomes. This approach supports broader adoption of minimally invasive surgery, particularly in low-income settings, where reducing procedural costs is critical.

## Introduction

With the global trend toward minimally invasive surgical techniques, video-assisted thoracoscopic surgery (VATS) has increasingly been adopted as an alternative to traditional thoracotomy. VATS is now a well-established technique worldwide, with various approaches including uniportal and multi-portal methods.^1)^

Studies have demonstrated that VATS offers advantages over thoracotomy, such as shorter postoperative hospital stays, fewer readmissions, reduced pain, and improved physical function after 5 weeks.^2)^ Due to these benefits, along with its minimally invasive nature, VATS lobectomy is recommended as the standard approach for early lung cancer treatment.^3)^

Mechanical stapling devices are routinely used for bronchial and pulmonary vasculature sealing and ligation during anatomic lung resections. These devices are effective, reliable, and time-saving, and they help reduce postoperative air leakage and hospital stay durations.^4,5)^ Stapling is generally safer and quicker than suture closure for bronchial stump closure after lobectomy or pneumonectomy.^6)^ However, staplers are associated with high operating costs and can be challenging for ligating small pulmonary vessels (<7 mm).^7)^

While the stapler-less approach has shown feasibility and safety for laparoscopic ligation of various vessels, including pulmonary, splenic, and mesenteric vessels, there is a lack of studies on the efficacy and cost-effectiveness of suture-based ligation for bronchial and pulmonary vessels.^8)^ This case series aims to assess the safety, feasibility, and cost-effectiveness of a stapler-less technique in VATS by reporting on 7 patients who underwent uniportal VATS at our hospital.

## Materials and Methods

### Data collection

A retrospective review of the patients’ medical records was conducted. Seven patients who underwent a uniportal stapler-less VATS lobectomy or segmentectomy between March 2021 and February 2022 at Al-Makassed Hospital in Jerusalem were included in this study. All operations were performed by the same thoracic surgery team. The collected data included length of the operation, length of the intensive care unit (ICU) and hospital stay, intraoperative complications, quantity of blood loss, open thoracotomy conversion rate, postoperative complications, drain removal day, postoperative analgesic use, and the estimated operative cost.

### Inclusion criteria

Patient selection was based on a thorough preoperative assessment by the surgeon to ensure that the stapler-less uniportal VATS approach was feasible and safe. The inclusion and exclusion criteria for this study were as follows:

Inclusion criteria:

1. Patients with lung nodules, metastases, or bronchiectasis requiring surgical resection.
2. Patients with well-defined segmental or lobar anatomy suitable for dissection without mechanical stapling.
3. Individuals with adequate cardiopulmonary reserve, as determined by preoperative pulmonary function tests and echocardiography.
4. Cases where the pulmonary vessels and bronchial structures could be securely managed using suture ligation or polymer clips.
5. Patients in whom a minimally invasive approach was deemed appropriate after multidisciplinary surgical evaluation.

Exclusion criteria:

1. Although the fissure-less technique was implemented in some cases, patients with a *fully fused fissure* faced safety concerns due to extensive dissection.
2. Cases with severe fibrotic lung disease could compromise postoperative healing.
3. *Severe emphysematous lung disease* makes lung manipulation and suture closure unreliable.
4. Patients with *poor cardiopulmonary reserve*, as determined by preoperative functional assessments (e.g., severely reduced ejection fraction or significant hypoxia), are at increased risk.
5. Emergency cases requiring rapid hemostasis are those where a stapler-based approach would be preferable.

Patients who met the inclusion criteria underwent a comprehensive preoperative evaluation, including CT imaging, pulmonary function testing, and echocardiography. Intraoperative feasibility was assessed by the surgeon, and in cases where unforeseen difficulties arose (such as extensive adhesions, fused fissure, or intraoperative bleeding), conversion to an alternative approach was considered.

This approach ensured patient safety while demonstrating the feasibility and cost-effectiveness of stapler-less uniportal VATS in select cases.

### Surgical approach and postoperative care

Under general anesthesia, a double-lumen tube was inserted and guided by a flexible bronchoscope for lung isolation. Patients were placed in the lateral decubitus position, with the bed maximally flexed and the breakpoint just above the superior iliac crest. The thoracic surgeon stood at the ventral side, and the first assistant was on the opposite side.

For major procedures, an arterial line was used for hemodynamic and arterial blood gas monitoring, while peripheral intravenous lines were employed for fluid and drug administration.

A 3 cm incision was made at the 5th intercostal space along the anterior axillary line, with a wound protector used. A 10-mm, 30° thoracoscope was inserted through this incision and positioned posteriorly. Two or 3 additional instruments, such as suction/irrigator devices, electrocautery, endoscopic graspers, or scissors, were introduced through the utility port.

The pleura was initially explored to check for adhesions, carcinosis, or abnormalities. The hilum was accessed, and the pulmonary artery and vein branches were identified, encircled, and ligated with 2-0 silk ligatures and polymer clips before being divided with scissors or an energy device. The bronchial branch was dissected, encircled, and divided with scissors, then closed with a continuous 3-0 poly-p-dioxanone (PDS) suture.

The segmental plan was cut using a diathermy or energy device in segmentectomy cases, and wedge resections were performed with an energy device, with lung tissue sutured using 3-0 PDS sutures. An intercostal block was administered intraoperatively. Lung inflation and air leak tests were conducted under thoracoscopic control. Post-surgery, a chest tube was placed for pleural drainage. One patient required 2 chest drains and a thoracotomy due to bleeding from the right pulmonary artery (not the same site of clipping), which was converted to thoracotomy.

The chest tube was removed when drainage was <3 mL/kg, and patients were discharged the day after tube removal if the follow-up chest X-ray showed no signs of pneumothorax and no complications were observed.

## Results

### Patients’ demographics

Over the period of the study, 7 stapler-less uniportal VATS procedures were performed at our hospital. The mean age was 49 years. Three of our patients were female and 4 were male. Patients’ characteristics are summarized in **Table 1**.

**Table 1:** Patients’ characteristics

Patients’ number	Age	Gender	Smoking history	FEV1/FVC (%)	EF (%)	Comorbidities
1	23	Female	Non-smoker	87	Not done	Bronchiectasis
2	44	Female	Non-smoker	80	60	
3	60	Female	Smoker	82	60	IHD
4	37	Male	Smoker	77	60	
5	54	Male	Smoker	80	60	HTN
6	56	Male	Smoker	n/a	55	Rectal adenocarcinoma
7	70	Male	Smoker	80	55	Laryngeal squamous cell carcinoma

FEV1: forced expiratory volume in one second; FVC: forced vital capacity; EF; ejection fraction; IHD: ischemic heart disease; HTN: hypertension; n/a: not available.

### Preoperative assessment

All patients underwent a full history, physical examination, and basic laboratory investigations. All patients except 1 underwent preoperative echocardiography, which showed normal ejection fraction and valves, except for 1 who had mild anterior mitral leaflet prolapse.

Six of our patients had a suspicious nodule on the CT scan and were referred to the hospital for surgical intervention. Two of those were diagnosed with non-pulmonary malignancy with suspected metastasis: laryngeal squamous cell carcinoma and rectal adenocarcinoma. One patient had a history of bronchiectasis and was referred for segmentectomy. The operation included 2 lobectomies, 3 segmentectomies, a wedge resection, a metastasectomy, and 4 lymph node dissections or biopsies.

### Intraoperative details

The length of the operations ranged between 48 and 118 min, with a mean of 80 min. The estimated blood loss was minimal (10–100 mL) in 6 patients, while the 6th patient developed bleeding (1500 mL) from the main right pulmonary artery, and this is the case that was converted to open thoracotomy. The bleeding was caused by over-retraction of the hilar structures, and the location of the tear was not related to the site of clipping or suturing. The patient had no other intraoperative complications. Intraoperative details are summarized in **Table 2**.

**Table 2:** Intraoperative data

Patients’ number	Preoperative diagnosis	Operation	Operation time	ICU LOS	Hospital LOS	Blood loss (mL)	Conversion to thoracotomy
1	Left lower lobe bronchiectasis	Uniportal VATS staplerless LLL basal segmentectomy	58	2	4	10	Not converted
2	Mediastinal and hilar lymphadenopathy lung nodules	Uniportal VATS staplerless wedge resection of RML nodule and hilar LN biopsy	45	2	2	10	Not converted
3	Right upper lobe squamous cell carcinoma	Uniportal VATS converted to anterolateral thoracotomy for RUL lobectomy and LN	74	3	11	1500	Converted
4	LLL lung nodule, mediastinal lymphadenopathy	Left lateral basal segmentectomy (aim was lymph node biopsy)	67	2	3	100	Not converted
5	Left lower lobe mass	Uniportal VATS LLL lobectomy and LN dissection of stations 5 and 10	118	3	9	200	Not converted
6	LLL nodule on segment 6	Left uniportal VATS segmentectomy	110	2	2	10	Not converted
7	RLL lung nodule	Right uniportal VATS staplerless RLL metastasectomy	48	2	6	100	Not converted

ICU: intensive care unit; L.N: lymph nodes; LLL: left lower lobe; LOS: length of stay; RLL: right lower lobe; RML: right middle lobe; RUL: right upper lobe; VATS: video-assisted thoracic surgery.

### Postoperative course

All of the 7 patients were in stable general condition with no acute complications or bleeding. Patients were extubated on the same day of the operation and transferred to the ICU for close observation, which is standard after VATS at the hospital. The mean ICU stay was 2.28 days. The patients received and responded to paracetamol and metamizole for pain relief. Serial X-rays were done for follow-up, and in most patients, they showed well-inflated lungs with no evidence of effusion or pneumothorax. One patient developed surgical emphysema and prolonged air leak, for which an additional chest drain was inserted on postoperative day 6 and removed 4 days later.

Chest drains were removed after a mean of 3.25 days for those inserted in the operating room. Only the patient in whom there was a conversion to thoracotomy was kept with an anterior chest drain after discharge. All patients were discharged in good general condition and were hemodynamically stable, with wounds that were clean and showed no signs of infection. The shortest hospital stay among patients was 2 days for the 5th and 6th patients, while the longest was 11 days for the 3rd patient, with an average of 5.29 days for all patients.

## Discussion

VATS has been demonstrated to have several advantages when compared to classical open thoracotomy.^9)^ Multiple studies have shown that VATS lobectomy is a safe and feasible operation when performed by experienced surgeons, with similar survival and complication rates compared to open thoracotomy procedures.^10,11)^ Other studies indicate that VATS may reduce complication rates and improve survival compared to open thoracotomy.^9,12)^ In a non-randomized study comparing pulmonary function between VATS and open thoracotomy procedures, it was found that patients who underwent lobectomy using the VATS approach had better postoperative O_2_ saturation, PaO_2_, forced expiratory volume in 1 second, peak flow rates, and forced vital capacity compared to those undergoing thoracotomy.^13)^ Another study indicated that patients with limited pulmonary function would have better outcomes if the surgery is performed via VATS rather than traditional open techniques.^14)^

Studies have also shown that VATS segmentectomy, when compared to the open approach, is associated with equivalent oncologic results along with lower costs, shorter length of stay, and reduced morbidity. Other specific reductions in complications associated with the VATS approach include fewer infections, pulmonary and cardiac complications, and a shorter duration of chest tube use.^13,15,16)^

As the uniportal VATS approach provides a direct view of the surgical field and allows for a wide range of manipulation of surgical instruments, as well as being a less invasive technique than multiportal VATS, it has been spreading worldwide in major pulmonary resection surgeries.^16)^

Stapling in the management of lung parenchyma and bronchi was first reported in 1964. The routine use of staplers for lung resections has been progressively accepted, mostly to reduce operation time and improve surgical comfort.^17)^ In fact, the first published article about mechanical staplers was in 1959, when Americans became aware of it, and it described surgical stapler closure of bronchial stumps. Since then, staplers have substantially improved. Surgeons can now place many rows of staples and offer options for suturing tissues in a linear, circular, curved, or radial form.^17,18)^

Despite the widespread use of VATS, which usually requires many staplers, very few articles have been published about its complications in VATS surgeries.^17)^ However, stapling failure or other unfavorable stapling issues occasionally occur. Many problems, such as oozing or partial disruption of the stapler line, are considered normal and not usually reported. Other complications that are often reported include massive hemorrhage or total disruption of staplers requiring repair; however, these are not usually published.^17)^

The Central Japan Lung Cancer Surgery Study Group, which included 29 institutions, performed a retrospective multi-institution review on the adverse events of lung tissue, bronchial, and pulmonary vascular stapling. Intraoperative and postoperative complications of lung tissue and pulmonary vascular stapling included air leakage, laceration of adjacent lung tissue, stapling failure, oozing, bleeding, laceration of peripheral vessels due to compression, and technical injury to the vasculature at insertion. Regarding pulmonary vascular stapling, intraoperative adverse events were found to occur more frequently than postoperative adverse events and were more common in the pulmonary artery than in the pulmonary vein. Tissue brittleness, stapler swaying during stapling, mismatch between stapler and tissue thickness, and other issues were also recorded. Complications of bronchial stapling included bronchopleural fistula and bleeding from the chest wall, in addition to air leakage, stapling failure, and stapler defects.^19)^

Another study suggested that complications of stapling are more frequently encountered during VATS than open lung resection, and many factors can suggest the cause. The first cause might be the difficulty of estimating tissue thickness due to a lack of tactile feedback. The second is the mispositioning of the stapler tip, which can lead to excessive traction on the tissue. Finally, the small stapler tip makes it hard to load the lung tissue, which can result in tears of the parenchyma.^17)^

As both stapler-less and conventional VATS can have complications, taking the cost factor into consideration is critical, especially in developing and low-income countries with limited resources. It is worth noting that this can only be applicable in the hands of experienced thoracic surgeons in VATS, so that people with poor socioeconomic status do not get discouraged due to conventional VATS procedure costs.

According to a recent Chinese study published in 2020, the average length of operation for uniportal VATS segmentectomies was 115 min compared to a previous study that had an average of 153.36 ± 39.64 min, while the average operation time for uniportal VATS lobectomies was 159.2 ± 53.14 min or, in another study, 198 min. Our average length of operation time was shorter at 80 min, ranging from 48 to 118 min. Average blood loss for uniportal VATS segmentectomies and lobectomies in the literature is 80 mL, which is similar to our patients (10–100 mL), except for 1 who was converted to open surgery. The average ICU stay was 2.28 days, with the median ICU length of stay for uniportal segmentectomies being 1 day (0–7 days). The length of postoperative stay for segmentectomies was 2–12 days, and for lobectomies, it was 6.78 ± 3.37 days, which is similar to our average of 5.29 days for all patients. The chest tube duration for segmentectomies ranges from 1–9 days and for lobectomies it is 5.04 ± 2.88 days on average. In our case, chest drains were removed after an average of 3.25 days, which is similar to stapler-less uniportal VATS.^20)^

According to our study, stapler-less uniportal VATS is a viable option in the hands of experienced surgeons, with a low conversion rate, no long-term complications, no postsurgical wound infections, no recurrence between March 2021 and February 2022, and a much lower average cost. In conventional VATS, around 5–10 staplers are usually used for each patient, and each stapler costs around $300 ($180–400) from major companies, while sutures are inexpensive and available in all hospitals around the world. Stapler-less VATS might be particularly helpful in developing countries with lower income, as this can lower the cost burden on patients and encourage them to undergo lifesaving procedures.

## Conclusion

This case series demonstrates that stapler-less uniportal VATS is a safe, feasible, and cost-effective alternative to conventional stapler-based techniques. With minimal complications and significantly reduced costs, this method could enable broader access to minimally invasive thoracic surgery, particularly in resource-limited settings. Wider adoption requires experienced surgical teams to ensure safety and effectiveness.

## Declarations

### Consent for publication

No individual person’s data, images, or videos are included in this manuscript. Therefore, consent for publication is not applicable.

### Ethics approval and consent to participate

This study was conducted in accordance with the ethical standards of Al-Quds University and Al-Makassed Hospital. Ethics approval was granted by the Institutional Ethics Committee of Al-Quds University (reference number: 249/REC/2022). Written informed consent was obtained from all participants prior to the study.

### Funding

No specific funding was received for this study.

### Data availability

The data that support the findings of this study are available from the corresponding author upon reasonable request.

### Author contributions

Data collection and analysis: RA, AA, RS, and MI. Study conception, research supervision, and manuscript preparation: FAA. Drafting and review of the manuscript: all authors. Approval of the final version: all authors.

### Disclosure statement

The authors declare that they have no competing interests.
